# Vasopressin-secreting neurons derived from human embryonic stem cells through specific induction of dorsal hypothalamic progenitors

**DOI:** 10.1038/s41598-018-22053-x

**Published:** 2018-02-26

**Authors:** Koichiro Ogawa, Hidetaka Suga, Chikafumi Ozone, Mayu Sakakibara, Tomiko Yamada, Mayuko Kano, Kazuki Mitsumoto, Takatoshi Kasai, Yu Kodani, Hiroshi Nagasaki, Naoki Yamamoto, Daisuke Hagiwara, Motomitsu Goto, Ryoichi Banno, Yoshihisa Sugimura, Hiroshi Arima

**Affiliations:** 1 Department of Endocrinology and Diabetes, Nagoya University Graduate School of Medicine, Nagoya, 466–8550 Japan; 2 Department of Physiology, Fujita Health University, Toyoake, 470–1192 Japan; 3 Laboratory of Molecular Biology and Histochemistry, Fujita Health University Institute of Joint Research, Toyoake, 470–1192 Japan

## Abstract

Arginine-vasopressin (AVP) neurons exist in the hypothalamus, a major region of the diencephalon, and play an essential role in water balance. Here, we established the differentiation method for AVP-secreting neurons from human embryonic stem cells (hESCs) by recapitulating *in vitro* the *in vivo* embryonic developmental processes of AVP neurons. At first, the differentiation efficiency was improved. That was achieved through the optimization of the culture condition for obtaining dorsal hypothalamic progenitors. Secondly, the induced AVP neurons were identified by immunohistochemistry and these neurons secreted AVP after potassium chloride stimulation. Additionally, other hypothalamic neuropeptides were also detected, such as oxytocin, corticotropin-releasing hormone, thyrotropin-releasing hormone, pro-opiomelanocortin, agouti-related peptide, orexin, and melanin-concentrating hormone. This is the first report describing the generation of secretory AVP neurons derived from hESCs. This method will be applicable to research using disease models and, potentially, for regenerative medicine of the hypothalamus.

## Introduction

The neuroendocrine system, composed of the hypothalamus and pituitary gland, plays essential roles in regulating body temperature, reproductive behavior, and food and water intake^1,2^. AVP is synthesized in the magnocellular neurons of the paraventricular nucleus (PVN) and the supraoptic nucleus (SON) of the hypothalamus and is released into the systemic circulation from the posterior pituitary to act on V2 receptors in the kidney to promote the reabsorption of free water^1,2^.

Deficient AVP release leads to a disorder called central diabetes insipidus (CDI). Patients with CDI usually manifest polyuria as well as polydipsia, and water intake can reach 10 L/day^3^. CDI patients are treated with desmopressin, an analogue of vasopressin. However, the frequency of hyponatremia with desmopressin is not uncommon^4^. Furthermore, if CDI is accompanied by dysfunction of osmoreceptors, patients can become adipsic and show life-threatening dehydration^3^. To overcome these problems, regenerative medicine is warranted.

An *in vitro*-model of the human hypothalamus would help the study of its development, investigation of hypothalamic disease pathophysiology, and pharmaceutical screening for new drugs against hypothalamic diseases. The establishment of an *in vitro*-model of the hypothalamus is, therefore, essential. Several reports have shown differentiation of hypothalamic neurons from human pluripotent stem cells^5–7^. Merkle *et al*. demonstrated the differentiation of AVP neurons from human pluripotent stem cells, although the efficiency of differentiation was very low and AVP secretion was not demonstrated^6^. Thus, improvement in hypothalamic neuron induction is necessary to enable their use as a source for regenerative medicine or for the pathological analysis of CDI.

In this study, we aimed to establish an *in vitro*-method for the functional differentiation of AVP neurons that secret AVP using human embryonic stem cells (hESC).

Our strategy was to optimize each step of hypothalamic development with reference to murine embryology (Fig. S1A,B). AVP neurons differentiate *in vitro* from “rostral and dorsal” hypothalamic progenitors^8^; therefore, we focused on the generation of individual ventral and dorsal hypothalamic progenitors *in vitro*.

## Results

### Differentiation into RAX+ hypothalamic progenitors

**Figure 1:**
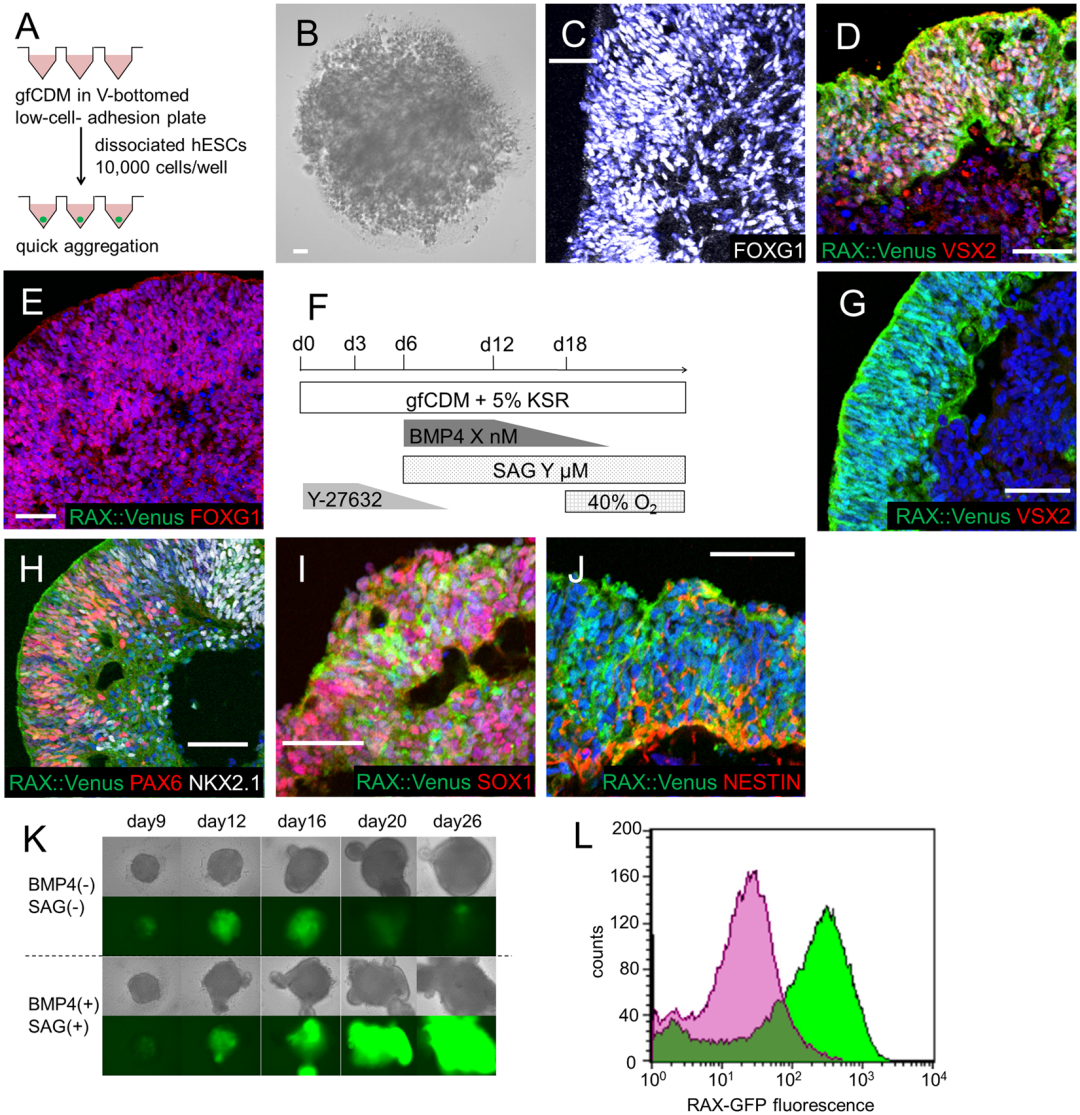
Differentiation into RAX+ hypothalamic progenitors. (**A**) Schematic of 3D culture (SFEBq) start conditions. (**B**) Failure of aggregation in SFEBq with gfCDM without KSR. (**C**) FOXG1 (white) expression in day 20 aggregates cultured with gfCDM containing KSR. FOXG1 is a telencephalic marker. (**D**) Induction of neural retina in day 30 aggregates in SFEBq with gfCDM + KSR and with BMP4 treatment. RAX::Venus (green), VSX2 (red). (**E**) Increased FOXG1 expression in day 20 aggregates treated with SAG in gfCDM + KSR. RAX::Venus (green), FOXG1 (red). (**F**) Culture protocol for induction of hypothalamic progenitors. (**G**–**J**) Immunostaining of day 30 aggregates with both BMP4 and SAG treatment in gfCDM + KSR. (**G**) RAX::Venus (green)+, VSX2 (red) −indicated hypothalamic progenitors. (**H**) Mixed pattern of dorsal (PAX6+) and ventral (NKX2.1+) hypothalamic precursors. RAX::Venus (green), PAX6 (red), NKX2.1 (white). (**I**,**J**) Expression of neural ectoderm markers. RAX::Venus (green), SOX1 (**I**, red) and NESTIN (**J**, red). (**K**) Time courses of RAX::Venus expression. Upper panels: bright field view, lower panels: fluorescence views of RAX::Venus+ cells. (**L**) FACS-analysis of day 20 aggregates. Over 80% of cells were induced to RAX+ hypothalamic progenitors. Green: BMP4 (+)/SAG (+), Purple: BMP4 (−)/SAG (−). For all relevant panels, nuclear counter staining: DAPI (blue), scale bar: 50 μM.

Wataya *et al*. reported a method for differentiating hypothalamic neurons from mouse embryonic stem cells (mESCs)^8^. Based on this report, we employed a three-dimensional floating culture, a serum-free floating culture of embryoid body-like aggregates with quick reaggregation (SFEBq)^9^, as our basic differentiation method (Fig. 1A). In the mESCs culture, Wataya *et al*. successfully induced AVP neurons using growth factor free chemically defined medium (gfCDM), which contained minimal exogenous inducing signals.

Rax is specifically expressed in rostral hypothalamic progenitors and the neural retina^10^. We used a RAX::Venus reporter hESC line^11^ to monitor hypothalamic differentiation in real time.

We first used gfCDM as a hESC-differentiation medium based on the report of Wataya *et al*.^8^; however, hESCs failed to form aggregates (Fig. 1B). Under these conditions, most human ESCs in SFEBq died within several days. When we added a small amount of knock out serum replacement (KSR)^12,13^ into gfCDM, hESCs successfully aggregated, but they began to express FOXG1^14^, indicating that they were telencephalic progenitors (Fig. 1C)^15^.

The concentration gradients of sonic hedgehog (SHH; secreted from the notochord and located in the ventral part of neural tube in chordates)^16–18^ and bone morphogenetic protein (BMP; secreted from the dorsal ectoderm)^19^ are essential for the development of the neural tube in vertebrates. Therefore, we next analyzed the effects of these signals on hESC culture. Addition of BMP4 or smoothened agonist (SAG, which induces Shh pathway activation) to the culture medium caused differentiation of hESC-aggregates into neural retina (VSX2+/RAX::Venus+; Fig. 1D)^20,21^ and telencephalic progenitors (FOXG1+/RAX::Venus−; Fig. 1E), respectively. When both factors were combined (Fig. 1F), hESC-aggregates differentiated into hypothalamic precursor cells (RAX::Venus+/VSX2−; Fig. 1G). These results suggest that SAG has the ability to shift the positional information from neural retina (Fig. 1D) toward hypothalamic precursors (Fig. 1G). In these aggregates, dorsal hypothalamic precursors (RAX::Venus+/PAX6+/NKX2.1−/VSX2−) and ventral hypothalamic precursors (RAX::Venus+/NKX2.1+/PAX6−/VSX2−) existed in a mixed pattern (Fig. 1H). Neural ectoderm markers (SOX1 and NESTIN)^22,23^ were co-expressed with RAX::Venus (Fig. 1I,J). These results indicate that hypothalamic precursor-like cells were successfully induced from hESCs. Under the hypothalamic precursor-inducing culture conditions using both BMP4 and SAG, time-course analyses showed that RAX expression increased from day 20 but that this increase was not produced under telencephalic-inducing conditions (Fig. 1K). Over 80% of cells were induced to RAX+ hypothalamic progenitors (Fig. 1L), suggesting that these inducing conditions were highly efficient.

### Specific induction of dorsal or ventral hypothalamic progenitors

**Figure 2:**
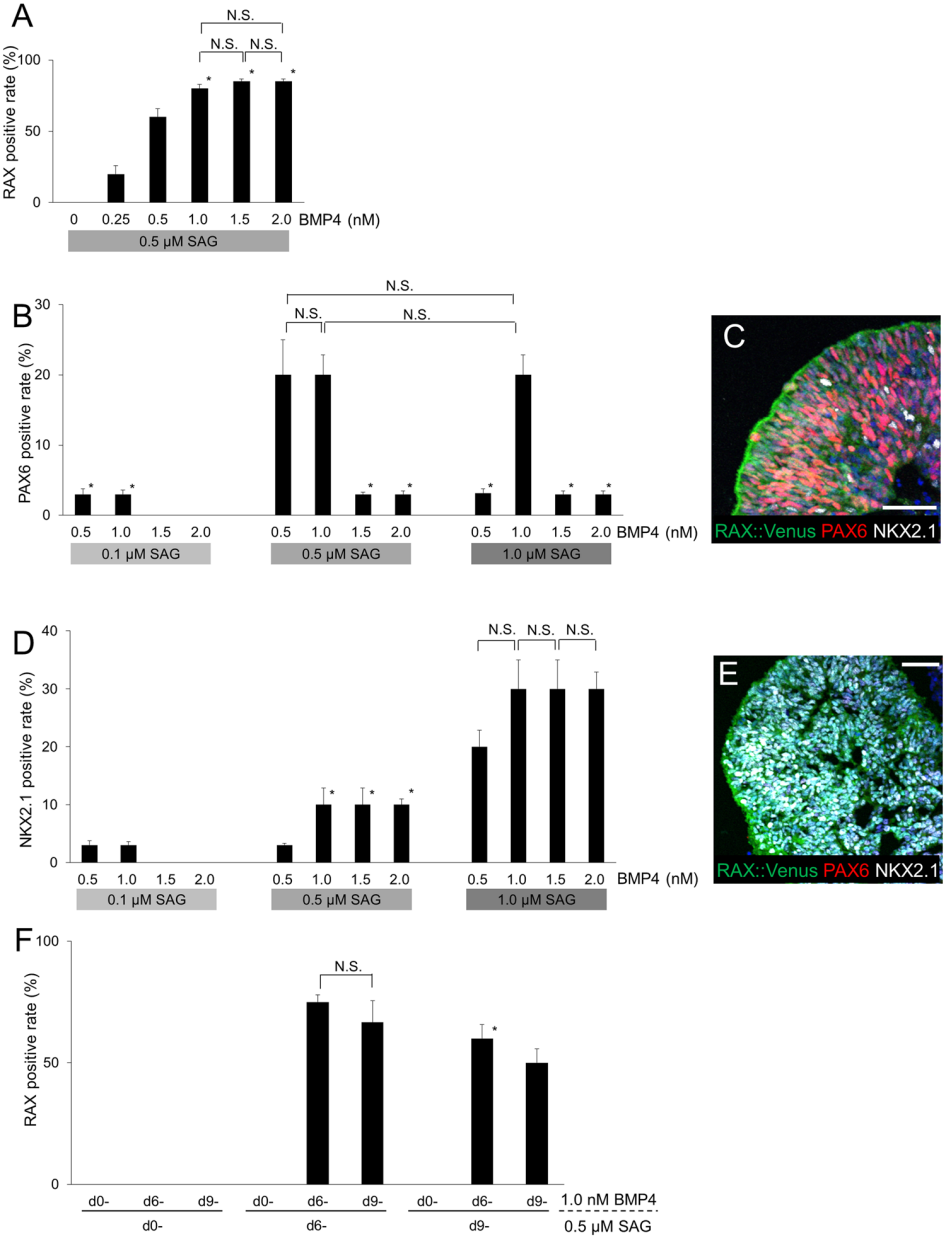
Specific induction for dorsal or ventral hypothalamic progenitors. (**A**) Effect of BMP4 concentration on RAX induction on day 30. n = 3, each column. *P < 0.05 vs BMP4 0.5 nM column. (**B**) Induction rates of PAX6+ dorsal hypothalamus in day 30 aggregates treated with the combination of BMP4 and SAG. n = 3, each column. *P < 0.05 vs BMP4 1.0 nM/SAG 0.5 μM column. (**C**) Immunostaining of a day 30 aggregate treated with 0.5 nM BMP4 and 0.5 μM SAG. RAX::Venus (green), PAX6 (red), NKX2.1 (white). (**D**) Induction rate of NKX2.1+ ventral hypothalamus in day 30 aggregates treated with the combination of BMP4 and SAG. n = 3, each column. *P < 0.05 vs BMP4 1.0 nM/SAG 1.0 μM column. (**E**) Immunostaining of a day 30 aggregate treated with 1.5 nM BMP4 and 1.0 μM SAG. RAX::Venus (green), PAX6 (red), NKX2.1 (white). (**F**) Altering the timing of application of BMP4 and SAG. *P < 0.05 vs BMP4/SAG treatment both from day6 column. For all relevant panels, nuclear counter staining: DAPI (blue), scale bar: 50 μM. Error bars represent s.e.m.

During development, RAX+ hypothalamic precursor cells become committed to segmenting into dorsal hypothalamus (RAX+/PAX6+/NKX2.1−) and ventral hypothalamus (RAX+/NKX2.1+/PAX6−) (Fig. S1B). We found that the concentration of BMP4 suitable for RAX induction was between 0.5–2.0 nM (Fig. 2A). Under these conditions, we examined the effects of SAG treatment at various concentrations. PAX6, which is a marker of the dorsal hypothalamus, was highly induced by a combination of BMP4/SAG with the concentrations of 0.5 nM/0.5 μM, 1.0 nM/0.5 μM, or 1.0 nM/1.0 μM (Fig. 2B). Many of the PAX6+ cells co-expressed RAX after this combination treatment (Fig. 2C). On the other hand, the expression of NKX2.1 (a ventral hypothalamic marker) was increased at a SAG concentration of 1.0 μM (Fig. 2D,E). Next, we tried alteration of timing of BMP4/SAG application (Fig. 2F). RAX was highly expressed when BMP4 was applied from day6 or day9 and SAG was from day6. Application of either BMP4 or SAG from day 0 resulted in no RAX expression. The late timing of BMP4 application (regardless of timing of SAG application) resulted in the highly expression of FOXG1 (telencephalon), and late SAG/early BMP4 application led to the highly expression of VSX1 (neural retina). From the above data, we determined suitable conditions for the individual induction of ventral or dorsal hypothalamus.

### Induction of AVP precursor cells

**Figure 3:**
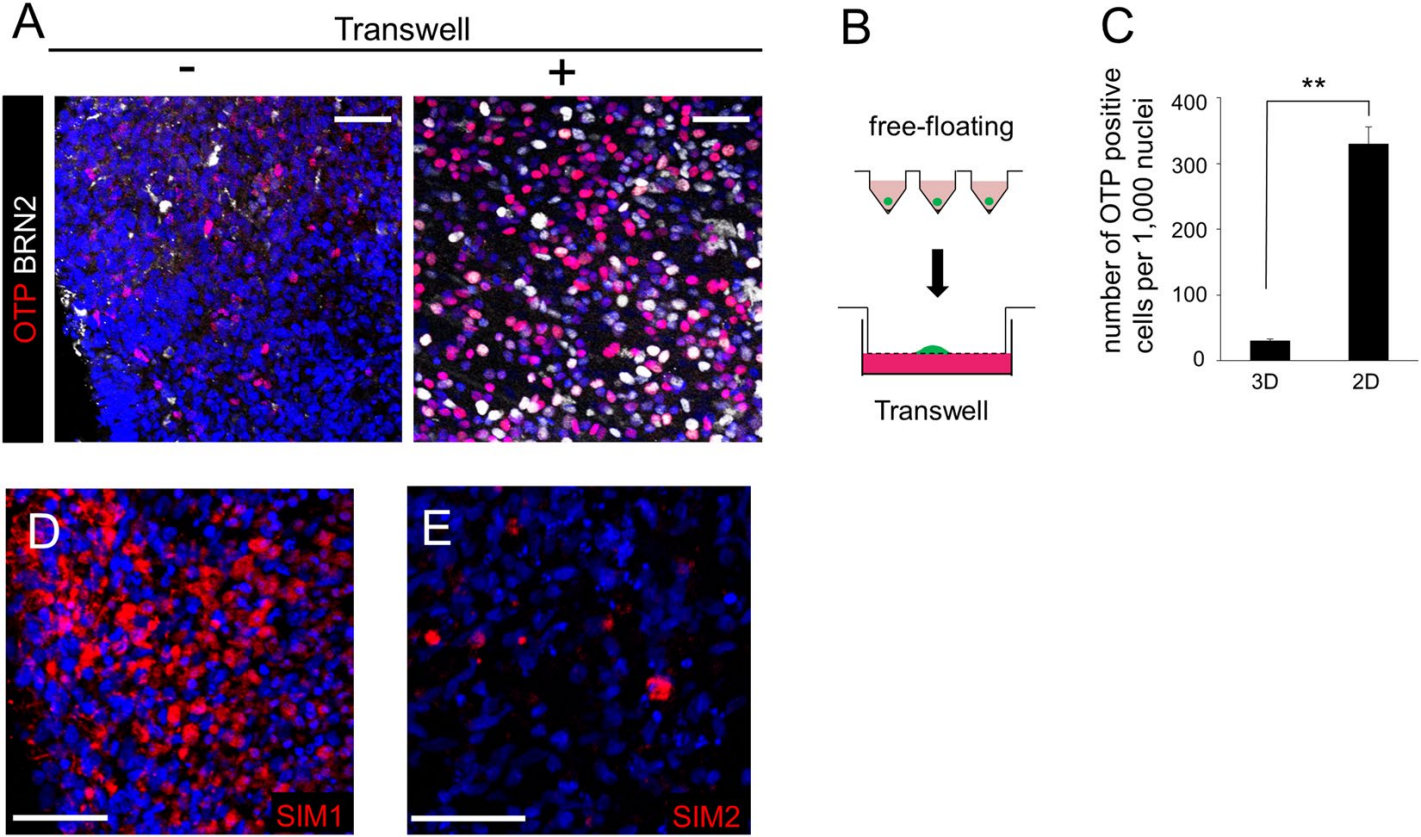
Differentiation into AVP precursor cells. (**A**) OTP and BRN2 staining in day 60 aggregates with (right panel) or without (left panel) transfer to Transwell. OTP (red), BRN2 (white). (**B**) Transfer of an aggregate from a V-bottom well to Transwell. (**C**) Number of OTP+ cells. 3D, continuous floating culture in V-bottom wells. 2D, gas-liquid interphase culture using Transwell. Approximately 10 times more cells were observed in the 2D cultures compared with the 3D cultures. n = 3. (**D**,**E**) SIM1 (**D**) and SIM2 (**E**) staining in day 60 aggregates. For all relevant panels, nuclear counter staining: DAPI (blue), scale bar: 50 μM. Error bars represent s.e.m. **P < 0.01.

AVP neurons are generated from the dorsal hypothalamus *in vivo* and we, therefore, checked whether AVP precursor cells appeared from hESC-derived dorsal hypothalamic (RAX+/PAX6+) aggregates. Even after long-term culture (60–80 days), only a few cells in floating aggregates expressed OTP or BRN2 (AVP precursor markers)^24–28^ (Fig. 3A, left panel). Following the precedent of Wataya *et al*.^8^, we then employed a gas-liquid interphase culture system using a Transwell porous filter in DMEM/F12 medium supplemented with glucose, N2 and B27 (DFNB)+ ciliary neurotrophic factor (CNTF) (Fig. 3B). As a result, over 30% of hESCs began to express OTP or BRN2 (Fig. 3A, right panel, 3C). Besides, SIM1 (hypothalamic precursor) and SIM2 (marker of diencephalon), were positive (Fig. 3D,E). These data indicate that these culture conditions were suitable for the induction of AVP precursor-like cells from hESCs.

### Generation of AVP-secreting neurons

**Figure 4:**
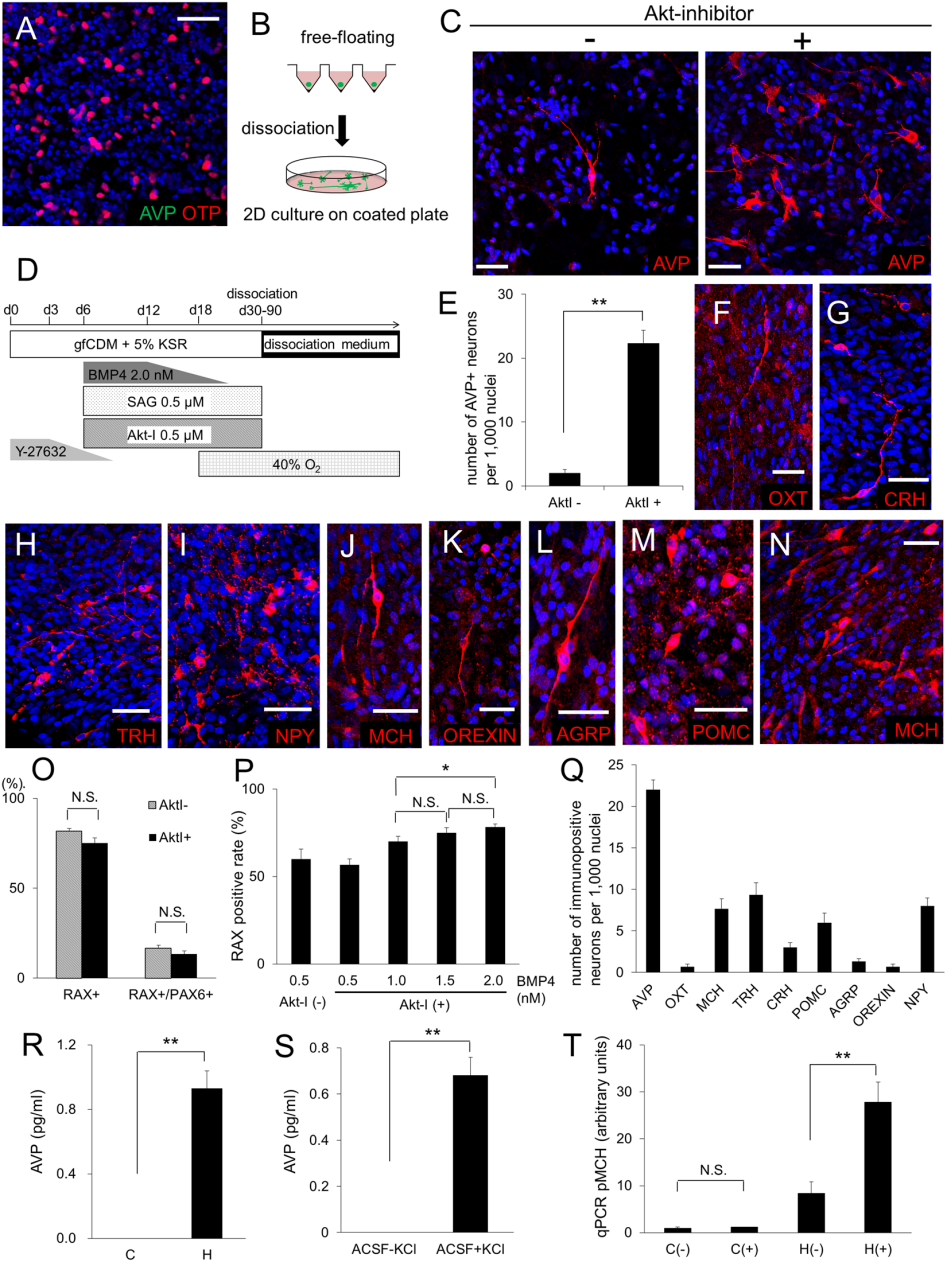
Generation of AVP-secreting hypothalamic neurons. (**A**) Immunostaining of a day 80 aggregate containing OTP+ cells. Few AVP+ cells were found in Transwell-cultured aggregates. AVP (green), OTP (red). (**B**) Schematic of 2D dissociation culture. Dissociated single cells from aggregates were attached onto coated glass-slips. (**C**) Effect of Akt-inhibitor on AVP induction. Immunostaining of day 150 aggregates with (right panel) or without (left panel) Akt-inhibitor treatment. AVP (red). (**D**) Schematic of the culture procedure for dorsal hypothalamic differentiation. (**E**) Number of AVP+ neurons with/without Akt-inhibitor treatment. n = 3. (**F**–**M**) Immunostaining for hypothalamic neurons in dissociation cultures treated with Akt-inhibitor. OXT (**F**, red), CRH (**G**, red), TRH (**H**, red), NPY (**I**, red), MCH (**J**, red), OREXIN (**K**, red), AGRP (**L**, red) and POMC (**M**, red). (**N**) MCH (red) immunostaining of an aggregate from ventral hypothalamus inducing conditions. (**O**) RAX and PAX6 positive rate with or without Akt-inhibitor treatment. Concentration of BMP4, SAG, and Akt-inhibitor was 1.5 nM, 0.5 μM, and 0.5 μM, respectively. RAX+/PAX6+ indicates double RAX/PAX6-positive cells. n = 3. (**P**) RAX positive rate with 0.5 μM SAG, 0.5 μM Akt-inhibitor, and 0.5-2.0 nM BMP4. n = 3. (**Q**) Number of immunopositive neurons per 1000 nuclei. n = 3. (**R**) AVP concentrations measured by radioimmunoassay in conditioned media of day 150 cells cultured with the Fig. 4D protocol. (**C**) control, using hESC-derived neocortex cells^38^. (**H**) hypothalamus, using dorsal hypothalamic neurons. n = 10. (**S**) AVP-release analysis measured by radioimmunoassay with or without high K stimulation on day 150. ACSF: artificial cerebrospinal fluid. n = 10. (**T**) pro-MCH analysis by quantitative PCR with or without nerve growth factor (NGF), dexamethasone (DEX) and lithium chloride (LiCl) stimulation on day 150.C: control, using non-hypothalamic differentiated aggregates. H: hypothalamus, using aggregates of ventral hypothalamic differentiation. (−): no stimulation. (+): with stimulation of NGF, DEX and LiCl. n = 6. For all relevant panels, nuclear counter staining: DAPI (blue), scale bar: 50 μM. Error bars represent s.e.m. *P < 0.05, **P < 0.01.

To induce AVP neurons from hESC-derived precursor cells, we continued the Transwell-culture, but only a few AVP+ cells were detected by day 60–80 (Fig. 4A). We next tried dissociation two-dimensional culture, which is a suitable method for culturing neurons. Floating aggregates were dissociated and mounted onto coverslips coated with Matrigel, poly-D-lysine and laminin (Fig. 4B). As a result, a few AVP neurons were detected on day 80–150 (Fig. 4C, left panel). For more efficient induction, we added Akt-inhibitor [Akt-I, which helps rostralization in hypothalamic development^8^] from day 6 together with BMP4 and SAG, to promote a more rostral positional identity (Fig. 4D). As expected, Neurophysin II was positive in day120 (Fig. S3A), and the number of hESC-derived AVP+ neurons was significantly increased (Fig. 4C,E). In addition to AVP neurons, we detected other hypothalamic neurons, such as cells positive for oxytocin (OXT), corticotropin-releasing hormone (CRH), thyrotropin-releasing hormone (TRH), melanin-concentrating hormone (MCH), orexin, neuropeptide Y (NPY), agouti-related peptide (AgRP)^29^ and pro-opiomelanocortin (POMC) (Fig. 4F–M). They were all positive for TUJ1^30^ (a neuronal marker; Fig. S2A), while there were also some non-neural glial cells (positive for GFAP, IBA1 and OLIG2; Fig. S3B–D)^31–33^. Furthermore, hESC-derived AVP+ cells seemed to be magnocellular neurons because CRH was not co-expressed in these cells (Fig. S2B), and large somas (about 40 × 20 μm, as seen *in vivo*) were predominant in *in vitro*-generated AVP neurons.

For optimization of culture conditions, we evaluated various concentrations of each induction factor. Treatment of Akt-I did not influence the induction rate of RAX+ cells or RAX+/PAX6+ cells (Fig. 4O). For BMP4, 2.0 nM was the most suitable concentration for RAX induction under Akt-I treatment (Fig. 4P). AVP+ neurons were best induced from the dorsal hypothalamic conditions (Fig. 4Q).

Next, to address the AVP secretion of *in vitro*-generated hypothalamic neurons, we stimulated long-term cultured neurons with potassium chloride (KCl), and measured AVP release using radioimmunoassays. AVP was detected in the medium of hypothalamic neurons (Fig. 4R), and the concentrations were further increased when the neurons were stimulated with KCl (Fig. 4S), suggesting that they were able to secrete AVP and react to stimulation.

MCH+ neurons were abundant from ventral hypothalamic aggregates by day 150 (Fig. 4N) and were more numerous compared with those from the dorsal induction protocol (Fig. S2E). qPCR analysis showed that treatment of these induced neurons with nerve growth factor, dexamethasone, and lithium chloride increased the expression of MCH (Fig. 4T), as seen *in vivo*^34^.

## Discussion

We aimed to establish a method to differentiate hypothalamic neurons from hESCs based on the protocol of Wataya *et al*.^8^. We succeeded in differentiating AVP neurons that also secreted AVP. The differentiation rate of AVP neurons seemed higher than that of the previous method^6^. The key for our efficient generation of AVP neurons is likely to result from specific induction of dorsal hypothalamic progenitors.

During early embryogenesis, the neural plate arises from thickened embryonic ectoderm and the hypothalamus originates from the rostral part of the neural plate. As the first step of neurogenesis, the frontal tip of the neural tube swells outwards. Next, the forebrain, midbrain and hindbrain are formed. The forebrain is subdivided into telencephalon and diencephalon, and finally the hypothalamus develops from the diencephalon (Fig. S1A). A key point for hypothalamic differentiation is its rostral position; it is differentiated from naïve neuroectodermal tissue. We first tried to induce rostral hypothalamic precursors, using RAX as a marker. As shown in Fig. 1, we treated hESC cultures with induction signals that are known to convey positional information, and we determined a combination of essential signals that lead to the rostral neuroectoderm differentiation. Figure 1L showed over 80% cells were differentiated into rostral neuroectodermal progenitors. On the other hand, a small number of non-hypothalamic differentiation was observed. For example, IBA1+ cells appear as shown in Supplementary Fig. 3C. IBA1 is known as not an ectodermal marker but a macrophage marker including microglia. However, very low positive rate of IBA1 (about 0.0006%) indicates that our method seems mainly to differentiate hypothalamic tissue.

Rostral hypothalamic progenitors consist of two groups, a dorsal part (PAX6+) and a ventral part (NKX2.1+). During development of the dorsal/ventral hypothalamus *in vivo*, it is well known that there are gradients of BMP and SHH signals in the developing hypothalamic area. Therefore, we tuned the concentration of BMP4 and SAG in the hESC cultures, and succeeded in individual induction of dorsal or ventral hypothalamic cells. As a result, OTP and BRN2, which are precursors of AVP neurons, were strongly expressed in hESC aggregates cultured with dorsal hypothalamic conditions, and finally, we achieved successful differentiation of AVP neurons. This is the first report of induced hypothalamic AVP neurons that secrete the hormone and react to KCl stimulation. We speculate that the key points of our differentiation method are accurate recapitulation of each developmental step and scrupulous optimization of differentiation efficiency.

It is difficult to clearly explain why the Transwell culture improved OTP+/BRN2+ hypothalamic differentiation. We speculate that it depends, at least partially, on O_2_ levels. When cultured in a hyperoxic (40% oxygen) condition from day 18, hypothalamic precursor cells were more highly expressed than in a normoxic (20% oxygen) environment. It is partially because, in a 20% O_2_ environment, cellular necrosis is likely to occur more frequently in the center of the aggregates than in 40% O_2_ condition (Supplementary Fig. 3M,N). It could be an important factor that there are no circulating systems such as blood vessels in hESC aggregates.

To obtain AVP+ neurons, the Transwell culture was not sufficient and additional dissociation two-dimensional culture was effective. We speculate that one of the reasons why AVP neurons were not efficiently induced by Transwell culture is insufficiency of extracellular matrix (ECM). Two-dimensional culture, which has scaffold for growth of neurite, is suited for differentiation of neurons and dissociation culture using ECM (e.g. laminin) is often adopted. ECM provides a microenvironment and affects nervous system development and function^35^.

There are a few limitations remaining in this report. The first is functional tests. We succeeded in generating AVP-secreting neurons, which reacted to depolarizing stimulation with KCl treatment. However, KCl is a very strong and non-specific stimulus, and more specific and precise functional tests will be necessary in future. Second, the number of human ESC lines tested in this study was small. We showed the successful differentiation using 2 cell lines, though the experiments with 3 or more lines are generally more desirable. There are not so many human ESC lines available in Japan, therefore, technical application to human iPSCs seems to be a next challenge.

In this study, we focused on BMP4, SAG and Akt-I as inducing molecules of hypothalamic neurons. However, there are also other signals that may influence development of the neural plate, such as WNT, retinoic acid, FGF and TGF. For example, the WNT pathway is known to affect anterior patterning, including the region that gives rise to the hypothalamus^36^. In addition, the concentration of WNT could affect the induction of neurons in the forebrain; lower WNT signaling induces the telencephalon, while higher WNT signaling causes differentiation of the : diencephalon, a part of which gives rise to the hypothalamus. Thus, optimization of the differentiation method with WNT or other signals remains an important objective for future research.

In summary, we established an induction method for AVP-secreting neurons from hESCs, through the specific induction of dorsal hypothalamic precursors. Our method may be beneficial for future studies of disease models, embryological investigations, development and screening of novel drugs, and regenerative medicine for CDI or other hypothalamic diseases.

## Materials and Methods

### hESC lines and maintenance culture of hESCs

hESCs were used according to the hESC research guidelines of the Japanese government. For the experiments shown, we used the VA22-N37 cell line^11^; however, for reproducibility, the results were confirmed using KhES-1 (KUIMSe001-A) and KhES-3 (KUIMSe003-A) cells (Fig. S4). VA22-N37 is a RAX::Venus reporter hESC line^11^ established based on KhES-1 (KUIMSe001-A) and they are biological replicates. Undifferentiated hESCs were maintained on a feeder layer of mouse embryonic fibroblasts (MEFs) inactivated by mitomycin C treatment in DMEM/F12 (D6421/Sigma) supplemented with 20% (vol/vol) KSR (lot No. 1517496/Invitrogen), 0.1 mM non-essential amino acids (11140-050/Invitrogen), 2 mM L-glutamine, 5 ng/mL recombinant human basic FGF (068-04544/Wako), and 0.1 mM 2-mercaptoethanol under an atmosphere of 2% CO_2_. For passaging, hESC-colonies were detached and recovered *en bloc* from culture dishes by treating them with 0.25% (wt/vol) trypsin and 0.1 mg/mL collagenase IV in PBS containing 20% (vol/vol) KSR and 1 mM CaCl_2_ at 37 °C for 8 min. The detached hESC clumps were broken into smaller pieces by gentle pipetting. The passages were performed at a 1:5 split ratio. When we performed SFEBq culture, small broken pieces of hESC clumps were incubated on gelatin-coated plate for 90 minutes to eliminate MEFs (Fig. S5).

### Three-dimensional free-floating culture of hESCs

For hypothalamic differentiation by SFEBq culture, hESCs clumps were dissociated to single cells in TrypLE Express (12605-010/Invitrogen) containing 0.05 mg/ml DNase I (11284932001/Roche) and 10 mM Y-27632 (034-24024/WAKO). Cells were quickly reaggregated using low-cell-adhesion 96-well plates with V-bottomed conical wells (Sumilon PrimeSurface plate; Sumitomo Bakelite), in differentiation medium (10,000 cells/well, 100 μl) containing 20 mM Y-27632, which inhibits dissociation-induced apoptosis. The Y-27632 concentration was reduced in a step-wise manner by replacing half the medium with medium without Y-27632, as in Fig. 1F.

The differentiation medium was gfCDM^8^ supplemented with 5% (vol/vol) KSR, 0.1 mM non-essential amino acids, 1 mM pyruvate, 0.1 mM 2-mercaptoethanol and half the medium volume was changed every other day.

On day 6, 0.5–4.0 nM BMP4 (5020-BP-010/R&D), 0.2–2.0 μM SAG (11914/Cayman Chemical), 1.0 μM Akt-inhibitor (124018/Calbiochem) were added when half of the medium volume was changed (final concentration was 0.25–2.0 nM, 0.1–1.0 μM and 0.5 μM, respectively). BMP4 concentration was maintained until day12 and reduced in a stepwise manner as in Fig. 1J, but SAG concentration was maintained. These medium conditions continued until day 30, 60, or 80.

From day 60, half of the medium volume was replaced with DFNB + CNTF medium^8^ every other day. Cell aggregates were maintained in free-floating conditions until day 80.

### Two-dimensional culture with Transwell

On day 60, aggregates were subjected to filter culture using a Transwell culture insert (3492/Corning) in DFNB + CNTF medium. A half of the medium volume was changed every other day.

### Dissociation culture of hESC aggregates

On day 60, cell aggregates were transferred as dissociated cells to Matrigel (354230/Corning), poly-D-lysine (354210/Corning) and Laminin (354232/Corning)-coated coverslips in 24-well plates with flat-bottomed columnar wells. Cell aggregates were dissociated by enzymatic digestion in Accumax (AM105/Funakoshi), Y-27632 and DNase I for 10 min at 37 °C, followed by mechanical trituration, pelleting and plated at a density of 200,000–300,000 cells/ml/well in dissociation medium, which contained DFNB, 10% FBS (SFBM30-2537/Equitech-Bio), BDNF (028-16451/WAKO), NT-3 (146-09231/WAKO) and LM22A-4 (SML0848/SIGMA), with 10 μM Y-27632. Half of the dissociation medium volume was changed every other day.

### Immunohistochemistry

Immunohistochemistry was performed as described with the primary antibodies described below. The antibodies were used at the following dilutions:

AVP (T-5048/guinea pig/1:2000/Peninsula), OTP (MS1535GS/guinea pig/1:2000/Takara), BRN2 (sc-6029/goat/1:500/Santa-Cruz), OXT (MAB5296/mouse/1:100/Millipore), AgRP (GT15023/goat/1:250/Neuromics), NPY (ab30914/rabbit/1:1000/abcam), Orexin (MAB763/mouse/1:500/R&D systems), MCH (M8440/rabbit/1:500/Sigma), CRH (sc-1759/goat/1:50/Santa-Cruz), POMC (ab14064/chicken/1:200/abcam), TUJ1 (PRB-435P/rabbit/1:2000/Covance), NKX2.1 (18-0221/mouse/1:500/Novocastra), Nestin (PRB-315C/rabbit/1:200/Covance), PAX6 (PRB-278P/rabbit/1:250/Covance), VSX2 (X1180P/sheep/1:500/Exalpha), TRH (HPA035595/rabbit/1:1000/Sigma), SOX1 (AB5934/chicken/1:500/Chemicon), OLIG2 (AB9610/rabbit/1:200/Millipore), GFAP (AB5804/rabbit/1:200/Chemicon), IBA1 (019-19741/rabbit/1:200/Wako), FOXG1 (AS3514/rabbit/1:3000/custom)^9^, RAX (1380RT/guinea pig/1:500/custom)^37^, SIM1 (sc-8714/goat/1:100/Santa-cruz), Human nuclear antigen (ab191181/mouse/1:100/abcam), Mouse MHC Class 1 H2 Db (ab25228/mouse/1:250/abcam), Neurophysin II (MABN856/mouse/1:100/Millipore), Copeptin (sc-7812/goat/1:100/Santa-cruz). Positive/negative controls of AVP, Neurophysin II and Copeptin are shown in Supplementary Fig. 3E–L.

To evaluate the rate of RAX, PAX6 and NKX2.1 expression in various culture conditions, we examined 10 aggregates per condition. We counted the number of all cells and all immunopositive cells of aggregates, and calculated the rate of expression of transcription factor. And we repeated more than three times.

### Vasopressin Release Analysis

Cultured hESCs were analyzed on day 150-180. Cultured cells from four wells were initially incubated with 0.3 ml per well of artificial cerebrospinal fluid (aCSF)^8^ for 10 min at 37 °C, and were then stimulated with high K-aCSF (100 mM KCl) for 30 min. The incubation solution (1.2 ml in total) was frozen and its AVP content was analyzed using a radioimmunoassay kit (Yamasa), which is used clinically in Japan. The quantification limit of Yamasa’s AVP kit is 0.4 pg/ml and the detection limit is 0.25 pg/ml. The data of correlation of control and measured AVP and the standard curve is shown in Supplementary Fig. 4G and Supplementary Table 1.

### MCH expression Analysis

Cultured cells were incubated with culture medium containing 50 ng/ml NGF, 1 μM dexamethasone, 20 mM LiCl for 12 hours^34^. After washing with PBS, MCH mRNA levels were analyzed by qPCR.

### Quantitative RT-PCR

Total RNA was purified using the RNAeasy kit (Qiagen) after treatment with DNase (Qiagen). Quantitative RT-PCR was performed using Power SYBR Green PCR Master Mix (Life Technologies) and the MX3000P system (Agilent Technologies). Data were normalized to *GAPDH* expression. The primers used were as follows: GAPDH, forward 5′-TGACCACAGTCCATGCCATC-3′, reverse 5′-GACGGACACATTGGGGGTAG-3′; pro-MCH, forward 5′-CTTTCAGAAGGAAGACACTGCAGAA-3′, reverse 5′-TCAGTGGCAGACCATGATTTAAGAA-3′.

### FACS sorting

Cells were counted on a FACS Vantage SE (BD) and the data were analyzed with FACS Diva software (BD). On day 20, cells were dissociated to single cells using Accumax, DNase I, and Y-27632 treatment and filtered through a Cell Strainer (BD Biosciences), and analyzed at room temperature. RAX::Venus+ cells and RAX::Venus− cells were gated by referring to scattered plots of the undifferentiated hESCs population to avoid cross-contamination.

### Statistical analyses

Statistical significance of two-group comparison was tested by Student’s t-test and more than of three groups was one-way ANOVA.

## Electronic supplementary material

Supplementary Information
