# Supplementary material for: Vasopressin-secreting neurons derived from human embryonic stem cells through specific induction of dorsal hypothalamic progenitors
Source: Sci Rep. 2018 Feb 26;8:3615. doi: 10.1038/s41598-018-22053-x (PMC5827757; doi:10.1038/s41598-018-22053-x)
Supplement: Supplementary file 1 — Supplementary Information [file 41598_2018_22053_MOESM1_ESM.pdf]

Supplementary information

Vasopressin-secreting neurons derived from human embryonic stem cells through specific induction of dorsal hypothalamic progenitors

Koichiro Ogawa, Hidetaka Suga, Chikafumi Ozone, Mayu Sakakibara, Tomiko Yamada, Mayuko Kano, Kazuki Mitsumoto, Takatoshi Kasai, Yu Kodani, Hiroshi Nagasaki, Naoki Yamamoto, Daisuke Hagiwara, Motomitsu Goto, Ryoichi Banno, Yoshihisa Sugimura, and Hiroshi Arima

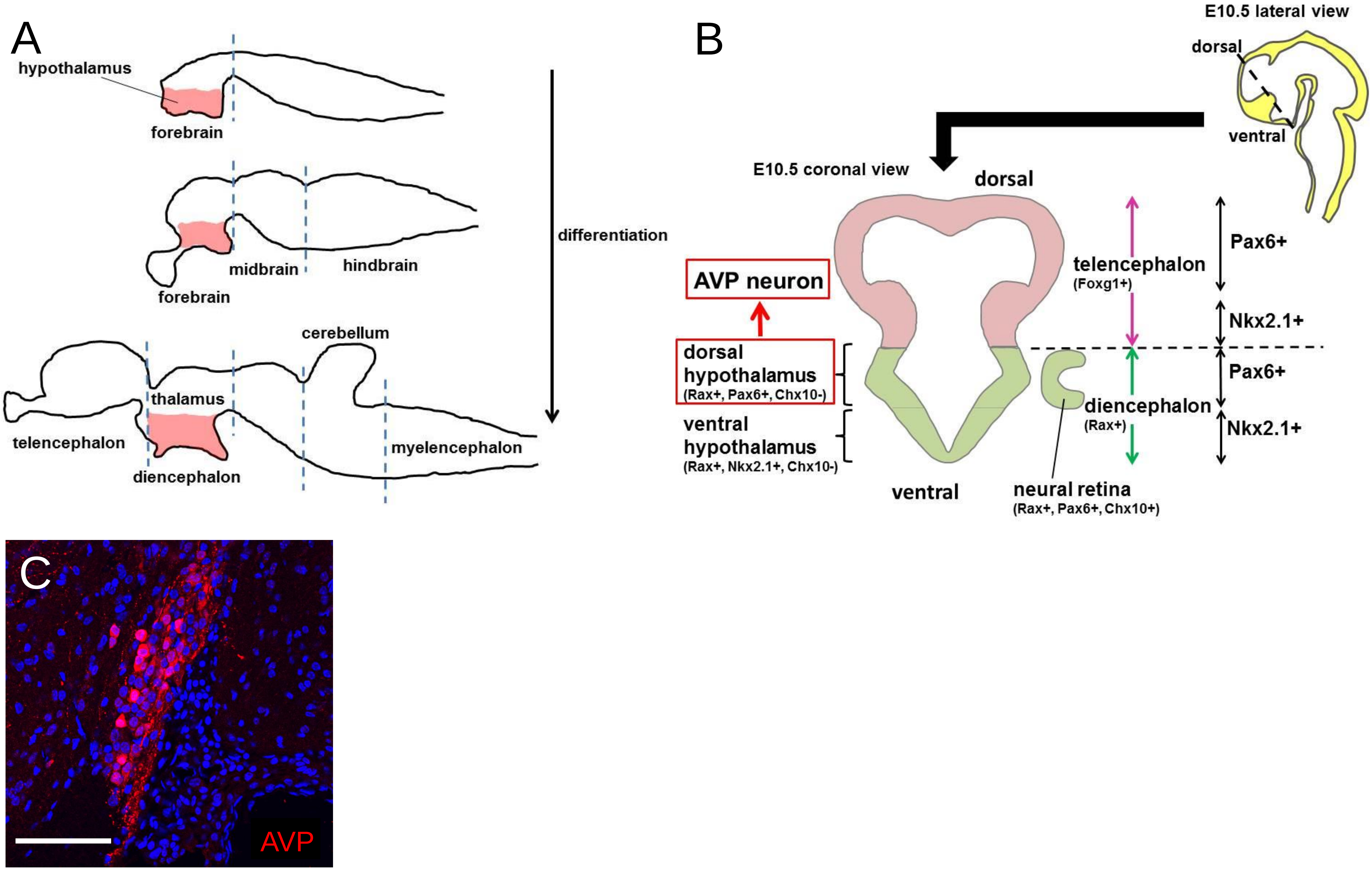

Supplementary Fig. 1. Schematic of embryonic mammalian brain development.
A) Schematic of the lateral view of neural tube development. The hypothalamus (red) is the most anterior and ventral neural structure.
B) Schematic of the anatomical subdivision and marker expression in the E10.5 mouse rostral forebrain. RAX expression is illustrated in green (modified from ref. 8).
C) AVP staning of the P1 mouse SON. Nuclear counter staining: DAPI (blue), scale bar: 50 μM.

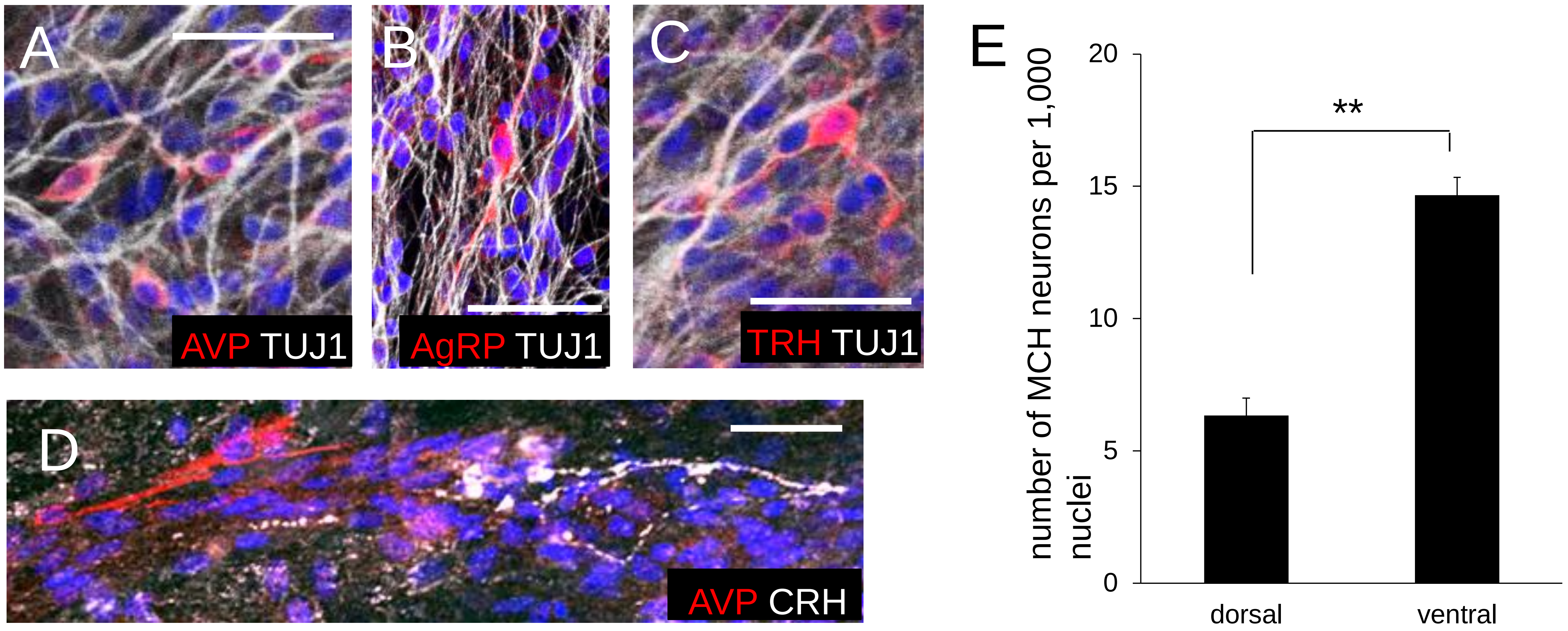

Supplementary Fig. 2. Immunohistochemistry of hypothalamic neurons.
A) AVP neurons co-expressed the neuron-specific marker, TUJ1. Other neuropeptide-immunopositive cells also co-expressed TUJ1 [e.g., AgRP (B), TRH (C)].
D) AVP+ neurons did not co-express CRH. This indicated the AVP+ cells as being magnocellular.
E) More MCH+ neurons were produced by the ventral hypothalamus induction protocol than by the dorsal protocol. n=3. Error bars represent s.e.m. **P < 0.01.
For all relevant panels, nuclear counter staining: DAPI (blue), scale bar: 50 μM.

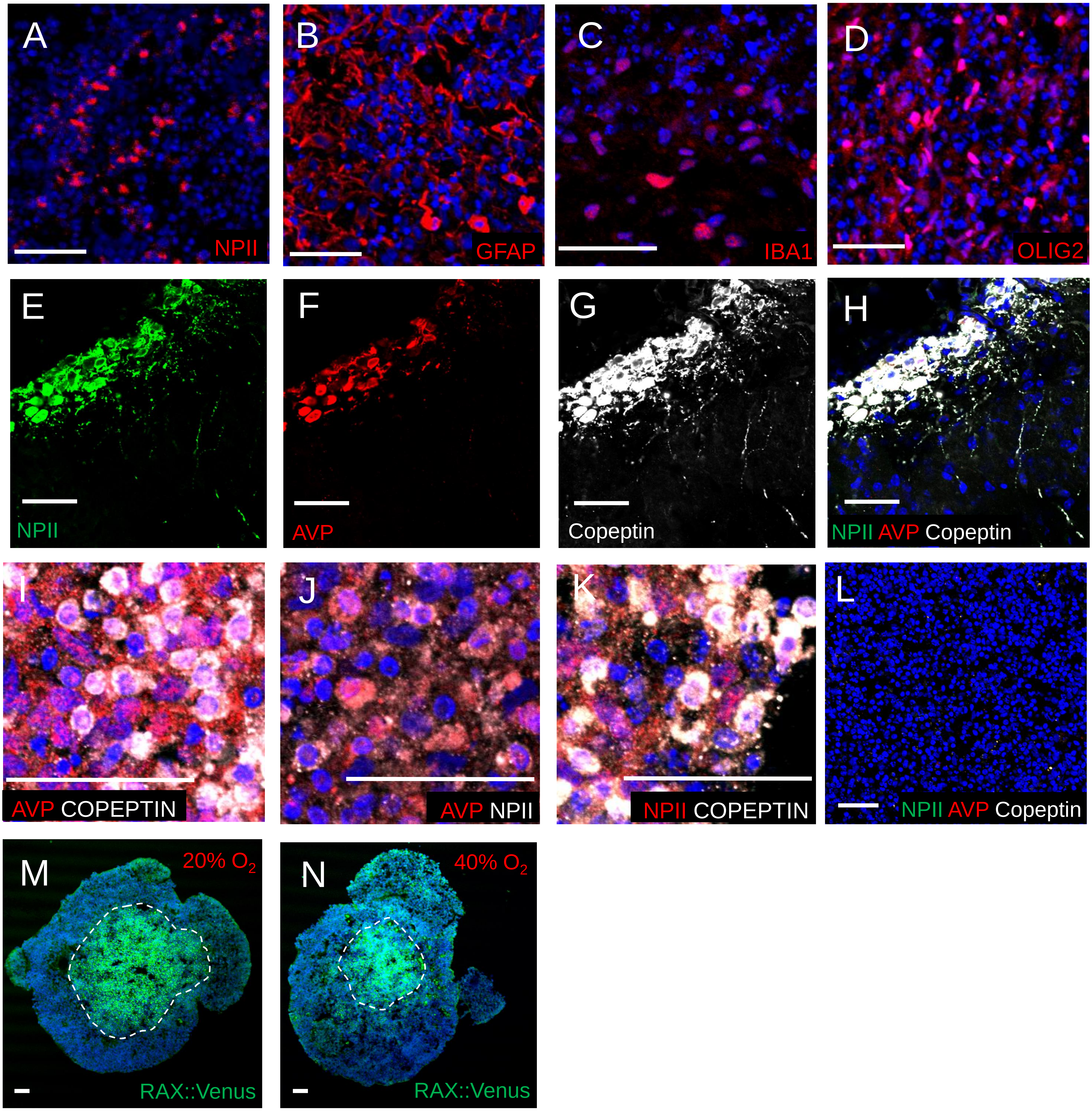

Supplementary Fig. 3. Immunostaining for AVP precursor and glial cell markers on day 120-150.
A) NPII (precursor of AVP neurons) staining.
B) - D) GFAP (B), IBA1 (C) and OLIG2 (D) are astrocyte, microglia and oligodendrocyte markers, respectively.
E) – H) Staining of mouse P4 hypothalamus, which is a positive control for Neurophysin II (NPII) (E), AVP (F), Copeptin (G), and merge (H).
I) – K) Co-localization of AVP, NPII and Copeptin in hESC-derived hypothalamic neurons.
L) Staining of hESC-derived neocortex cells, which is a negative control for NPII, AVP, Copeptin.
M), N) Comparison of cellular necrosis in the center of the aggregates in 20% with 40% O_2 condition of Day30 aggregates. The inside of the dotted circle is cellular necrosis region.
For all relevant panels, nuclear counter staining: DAPI (blue), scale bar: 50 μM

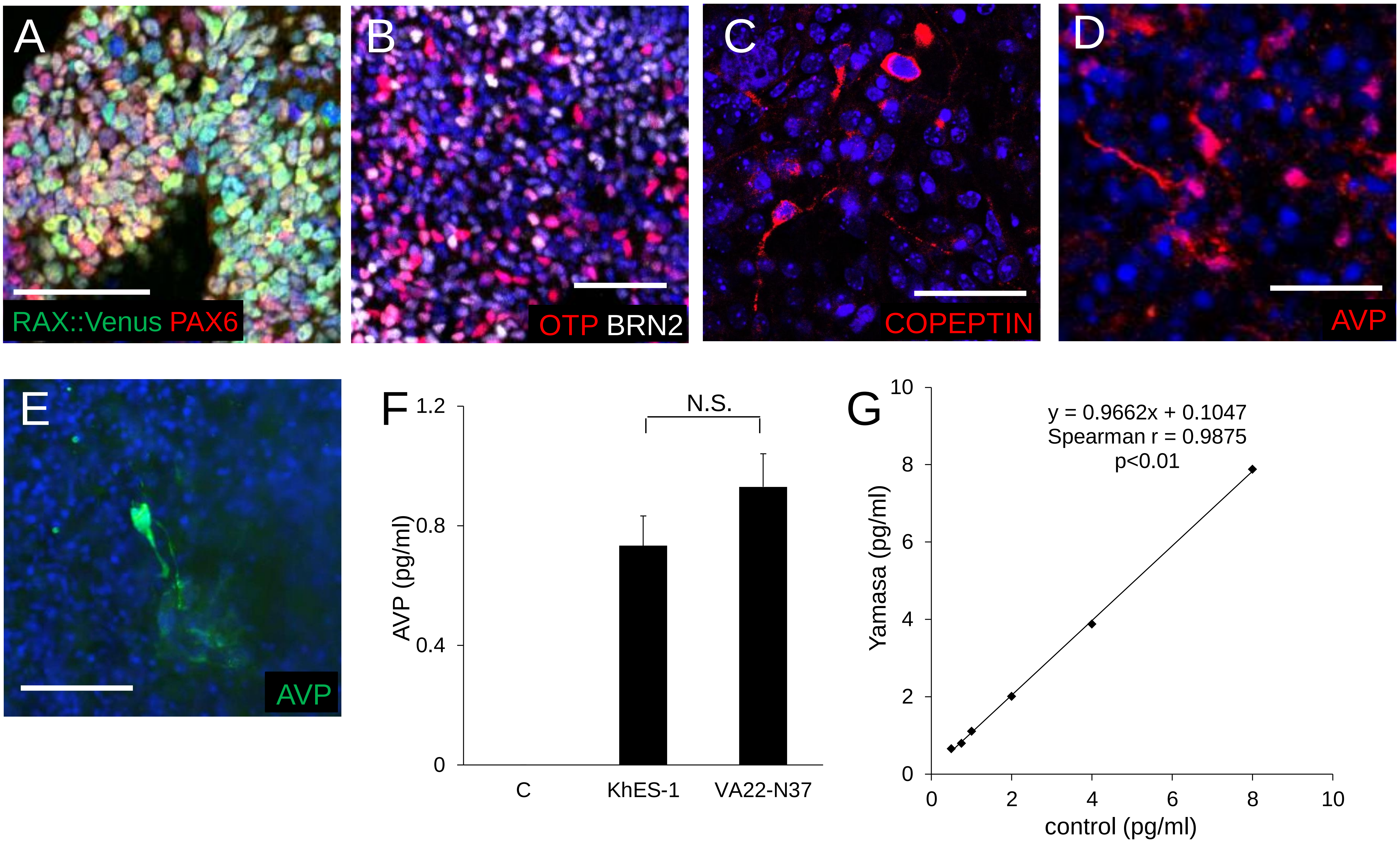

Supplementary Fig.4. Differentiation from KhES1 and KhES3.
A) – D) Differentiation of KhES1 into RAX+/PAX6+ dorsal hypothalamic progenitor (A), OTP+/BRN2+ AVP precursor (B), Copeptin+ AVP precursor (C), and AVP neurons (D).
E) Staining with AVP of KhES3-derived hypothalamic AVP neurons.
F) AVP concentrations measured by RIA in conditioned media of day 150 cells cultured with the Fig. 4D protocol. C: control, using hESC-derived neocortex cells (37). KhES-1: using dorsal hypothalamic neurons differentiated from KhES1. VA22-N37: using dorsal hypothalamic neurons differentiated from RAX::Venus cell line. n=10. Error bars represent s.e.m.
G) Significant Spearman correlation of control and measured AVP. Mean and s.e.m. of the examination are on Table.1

control (pg/ml)	Yamasa (pg/ml)	
	mean	s.e.m.
0.5	0.652	0.020
0.75	0.797	0.024
1.0	1.110	0.090
2.0	2.010	0.029
4.0	3.880	0.053
8.0	7.880	0.040

Supplementary Table.1
Data of AVP measured with Yamasa's RIA kit.

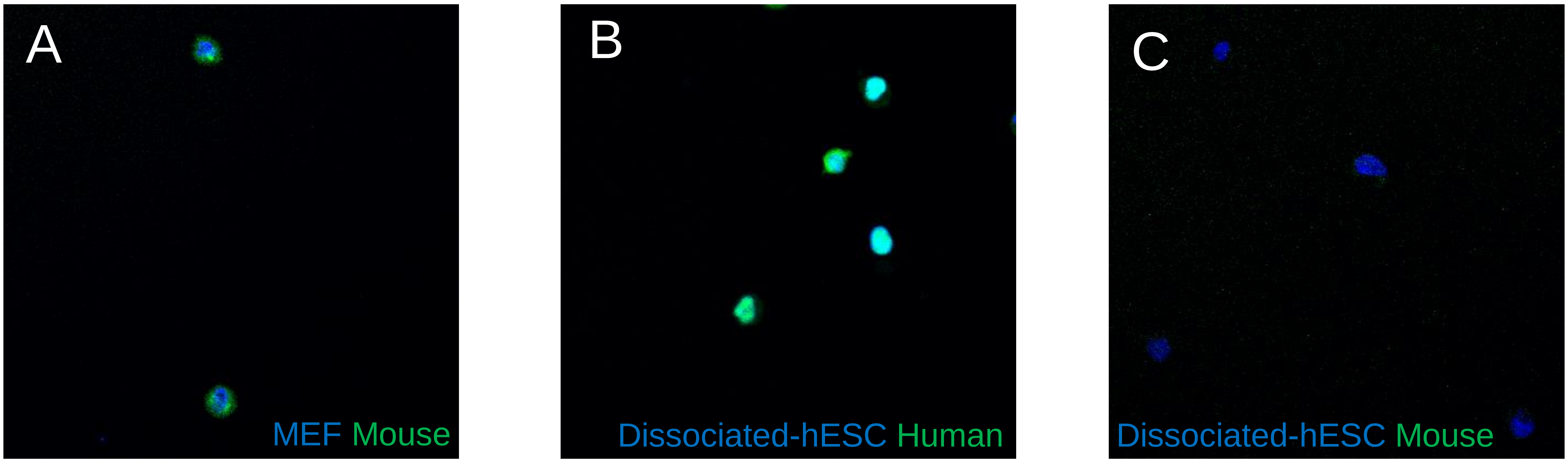

Supplementary Fig.5. MEF elimination in SFEBq method.
A) MEFs staining with anti-mouse antibody.
B), C) Staining of dissociated undifferentiated hESCs with anti-human antibody (B) or anti-mouse antibody(C).

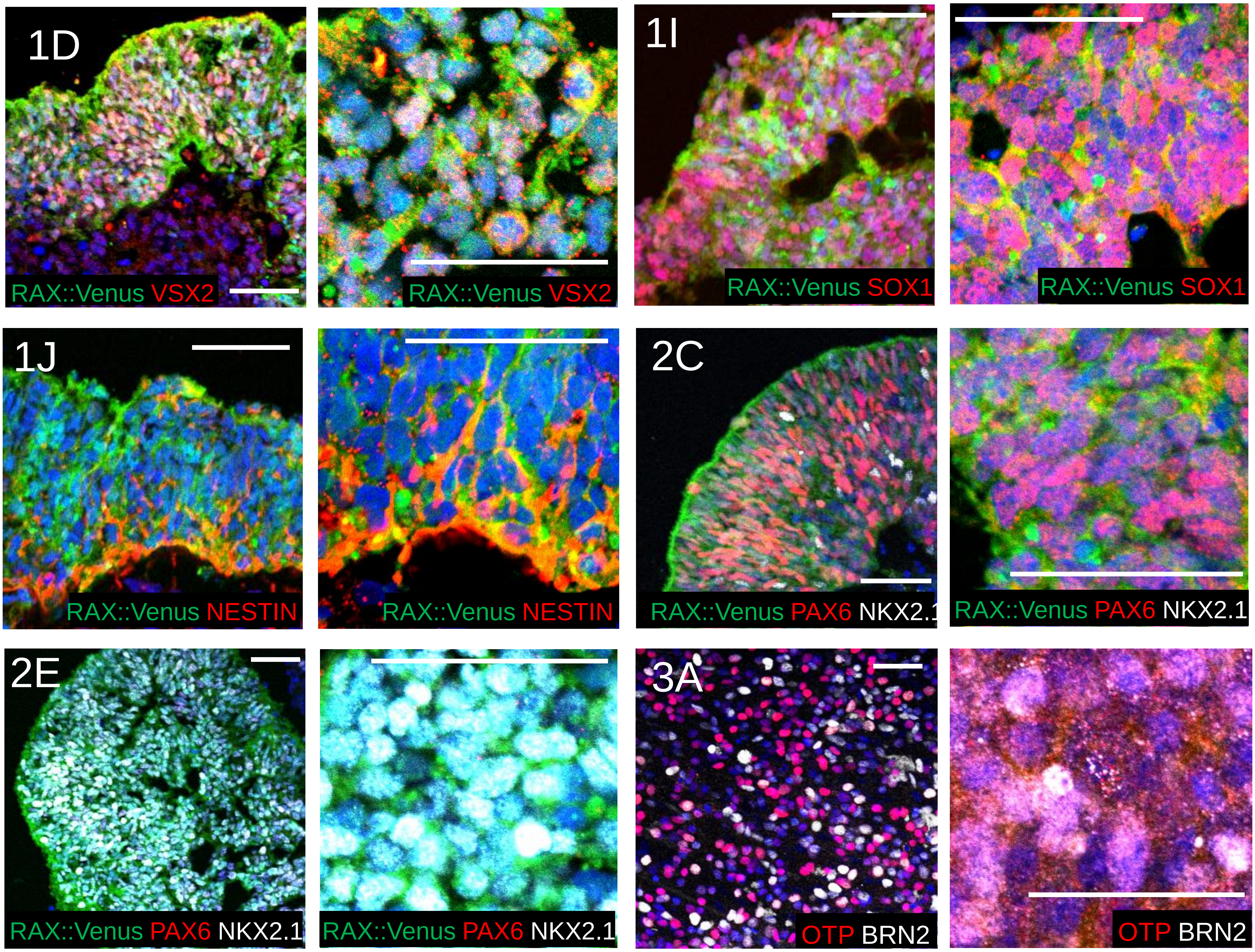

Supplementary Fig.6. High magnification images of co-localization of markers by immunostaining. The figure numbers shown in upper left corner correspond to the main figure number. The each magnified image is in the right panel.
